# A Novel Reinforcement Learning Approach for Spark Configuration Parameter Optimization

**DOI:** 10.3390/s22155930

**Published:** 2022-08-08

**Authors:** Xu Huang, Hong Zhang, Xiaomeng Zhai

**Affiliations:** School of Cyber Security and Computer, Hebei University, Baoding 071000, China

**Keywords:** Apache Spark, parameter optimization, deep neural network, Q-learning

## Abstract

Apache Spark is a popular open-source distributed data processing framework that can efficiently process massive amounts of data. It provides more than 180 configuration parameters for users to manually select the appropriate parameter values according to their own experience. However, due to the large number of parameters and the inherent correlation between them, manual tuning is very tedious. To solve the problem of tuning through personal experience, we designed and implemented a reinforcement-learning-based Spark configuration parameter optimizer. First, we trained a Spark application performance prediction model with deep neural networks, and verified the accuracy and effectiveness of the model from multiple perspectives. Second, in order to improve the search efficiency of better configuration parameters, we improved the Q-learning algorithm, and automatically set start and end states in each iteration of training, which effectively improves the agent’s poor performance in exploring better configuration parameters. Lastly, comparing our proposed configuration with the default configuration as the baseline, experimental results show that the optimized configuration gained an average performance improvement of 47%, 43%, 31%, and 45% for four different types of Spark applications, which indicates that our Spark configuration parameter optimizer could efficiently find the better configuration parameters and improve the performance of various Spark applications.

## 1. Introduction

Apache Spark [1] is a widely used open-source data analysis framework that utilizes resilient distributed datasets (RDDs) [2] to improve the efficiency of data processing and analysis while ensuring high fault tolerance and scalability. It provides a series of high-level components, including Spark streaming for real-time computing, Spark SQL for structured data processing, GraphX for graph computing, and MLlib for machine learning [3]. These components are applied by application developers to various fields, such as feature extraction [4], intrusion detection [5], and community discovery [6], and maintain good performance.

Apache Spark has more than 180 configuration parameters that users must manually adjust according to their application and cluster environment. Choosing the appropriate configuration parameters can not only significantly improve the performance of Spark applications and speed up the running time of Spark applications, but also improve the utilization of cluster resources. Unfortunately, some Spark’s configuration parameters are numerous, and their inter-relationship are also very complex [7]. Therefore, tuning configuration parameters is a very challenging task.

Manually tuning Spark configuration parameters is cumbersome and time-consuming, and requires developers to have a deep understanding of the Spark framework, which inspired our interest in the automatic tuning of Spark configuration parameters. Generally speaking, automatic Spark configuration parameter tuning consists of two components: the Spark application performance prediction model and the configuration-parameter-searching algorithm. Performance prediction models render the evaluation process efficient and practical instead of methods of repeatedly executing the application.

Gao et al. [8] employed SVM to build a model to predict the execution time of Spark applications. Rahman et al. [9] built a Spark performance prediction model using ANN. However, SVM, ANN, etc. are all shallow machine-learning methods that do not perform very well when the dataset has more noise or is complex and high-dimensional. In this paper, we introduce a deep-learning neural network to build a Spark performance prediction model. Deep-learning neural networks can gradually learn through multiple networks, extract complex and effective features, and have better prediction accuracy and generalization ability [10].

Recently, some methods based on reinforcement learning to find the optimal neural network model structure have been proposed. MetaQNN [11] applied the Q-learning algorithm to select various layers of a CNN that can automatically generate high-performance CNN architectures for a given task. BlockQNN [12] proposed a Q-learning algorithm to construct an optimal neural network block, and then applied the block-by-block stacking method to automatically construct the network. The above methods were mainly aimed at the construction of the neural network model structure. In this paper, we combine the Spark performance prediction model and the improved Q-learning algorithm to automatically search for optimal Spark configuration parameters in a discrete and limited parameter space. In particular, we make the following contributions in this paper:

- We designed and implemented a Spark configuration parameter optimizer that accurately predicts the execution time of Spark applications and gives the recommended configuration parameters. Experiments demonstrate that the performance of Spark applications using the recommended configuration parameters is significantly improved compared to the default configuration.
- From more than 180 Spark configuration parameters, we screened out key configuration parameters that had greater impact on the application to reduce the complexity of the model.
- We built a Spark application performance prediction model on the basis of a deep neural network, and verified the accuracy and usability of the model from multiple perspectives through experiments.
- On the basis of the Q-learning algorithm, we improved the optimal searching algorithm to explore unknown areas of the configuration parameter space, avoid local optimal solutions, and find a suitable parameter configuration for Spark applications in a short period of time.

The rest of this paper is organized as follows: Section 2 discusses the related work. Section 3 introduces the processing flow of the Spark configuration parameter optimizer and the method used in each section. Section 4 introduces the experiments and analyzes the experimental results. Section 5 concludes the paper.

## 2. Related Work

In recent years, the performance optimization of large data processing systems has been a hot-spot academic issue. Big data processing systems contain a large number of configuration parameters such as controlling parallelism, memory settings, and I/O behavior. Inappropriate parameter settings can lead to severe performance degradation [13]. Gounaris et al. [14] mapped their experience in a trial-and-error iterative improvement methodology for tuning parameters in arbitrary applications on the basis of evidence from a very small number of experimental runs. Due to the complexity between parameters, this method requires users to have rich experience in Spark configuration parameter tuning. This paper mainly studies the automatic optimization of Spark configuration parameters, that is, combining the Spark application performance prediction model and search algorithm to effectively traverse the configuration parameter space and give better configuration parameters for the application.

Research on Spark performance prediction models has always been a hot topic. Singhal et al. [15] proposed a gray box approach to estimate an application execution time on the Spark cluster for larger data using measurements on low-volume data in a small cluster. Huang et al. [16] proposed a cost optimization model for the Spark shuffle process that enables users to obtain the best compression configuration before application execution. Wang et al. [17] proposed a novel method for tuning the Spark configuration parameters on the basis of machine learning, and established a model based on binary and multiclassification. Islam et al. [18] modeled the Spark application completion time with respect to the number of executors and Spark application input or iteration. Chao et al. [19] built regression models for different stages during the run, and then built a regression model on the basis of the predicted time of each stage to predict the overall running time of the job. Cheng et al. [3] used Adaboost to build a set of stage performance models for Spark applications. At the same time, using projective sampling reduced the training samples of the performance prediction model and reduced the model overhead. Shah et al. [20] proposed an execution time estimation method called PERIDOT that estimated the dependencies between the internal stages of the application by analyzing the logs of two executions and then combined the knowledge of Spark’s data partitioning mechanism to derive an analytical model that estimated the execution time of an application on the basis of resource settings and input data size. However, the above performance prediction model cannot effectively deal with the problem of high-dimensional configuration parameters, and  accuracy decreased with the increase in configuration parameters.

Parameter search mainly aims to design a search algorithm to quickly find better configuration parameters from a larger configuration space. Wang et al. [17] adopted the recursive random search (RRS) algorithm to search a parameter space. Gu et al. [21] proposed a neural-network-based configuration tuning approach. In this approach, a neural network model was trained to predict the increase or decrease in configurations that determine the next search space. Ross [22] presented a sample-efficient, high-dimensional autotuner that used Bayesian optimisation with a directed-acyclic graph (DAG) surrogate model. Patanshetti et al. [23] proposed two search algorithms, grid search with finer tuning and controlled random search, which help in selecting those important parameters that affect the performance of Spark applications. We selected the next parameter to be changed by comparing the values in the Qtable, avoiding the search in the invalid configuration parameter space, and improving the search efficiency.

## 3. Methods

**Figure 1:**
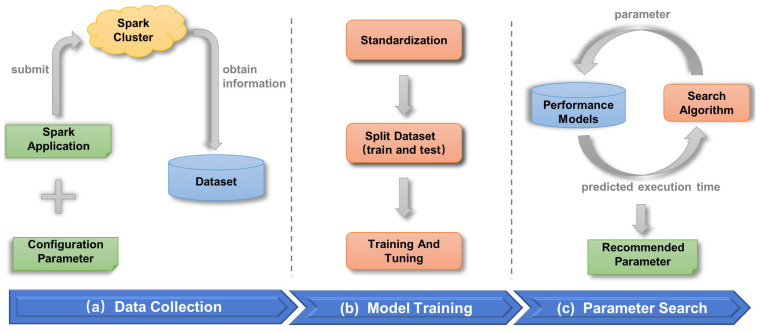
The framework of the Spark configuration parameter optimizer.

In this part, we introduce the Spark application configuration parameter optimizer in detail, which mainly consists of three phases: the collection and preprocessing of historical Spark application data; the training of a Spark application performance prediction model; and the search for better configuration parameters, corresponding to Figure 1a–c.

### 3.1. Data Collection and Preprocessing

#### 3.1.1. Parameter Selection

In Spark configuration parameter optimization, it is very important to choose an appropriate parameter space. A large number of parameter features increase the searching time, while few parameter features may lead to sub-optimal configuration. In the Spark parameter list, some parameters (such as spark.app.name) have no influence on the performance of the application, which are removed directly. Lastly, on the basis of theories [24,25] and experience [26], we selected 16 parameters that had the greatest impact on the performance of the Spark system. These parameters mainly covered the allocation and use of available cluster resources (CPU, memory, and disk), data transmission and compression, scheduling, etc. Table 1 lists the name, function, default value, and value range of each configuration parameter on the basis of our Spark cluster.

#### 3.1.2. Data Preprocessing

In the data collection and preprocessing phase, we applied Hibench [27] to generate the experimental data to be processed by Spark applications, and then randomly generated parameter sets P from configuration parameter space D. The representation is as follows:

(1) $$P=\left\{p_{i}\in D|1\leq i\leq M\right\}$$

(2) $$C=\left\{c_{ij}\in p_{i}|1\leq i\leq M,1\leq j\leq N\right\}$$

where *M* represents the number of parameter combinations, *N* denotes the number of parameter types in each parameter combination, $p_{i}$ represents the *i*-th parameter set obtained by sampling, and $c_{ij}$ represents the *j*-th parameter value in the *i*-th parameter set.

Lastly, we executed the Spark application with each parameter set on Yarn mode on our Spark cluster, and recorded the running time of the Spark application with different parameter sets to generate the basic history dataset for training in Equation (3).

(3) $$T=\left\{t_{1},t_{2},t_{3},\ldots ,t_{i}|1\leq i\leq M\right\}$$

where $t_{i}$ represents the Spark execution time under the *i*-th parameter set. Since the structure and format of each Spark configuration parameter are inconsistent, the value of each configuration parameter is normalized before model training by Equation (4):

(4) $$c_{ij}=\frac{\left(c_{ij}-\mu_{j}\right)}{\sigma_{j}}$$

where $c_{ij}$ is the original configuration parameter value, $\mu_{j}$ is the mean value of this type of configuration parameter, and $\sigma_{j}$ is the standard deviation of this type of configuration parameter. Standardization scales the value of each configuration parameter to the same numerical range, ensuring that the configuration parameters are at the same level, thereby improving the comparability of different configuration parameters.

### 3.2. Performance Prediction Model

After standardizing the base dataset for the Spark application, we generated a dataset for model training. To improve the efficiency of searching for optimal parameters, we used a performance model to evaluate the actual performance of the Spark application. In general, the performance of a Spark application [28] can be expressed as in Equation (5):

(5) $$Perf=Func\left(app,input,rsrc,config\right)$$

where Perf represents the running performance of the application, Func represents the performance evaluation function, app is the Spark application, input represents the input data, rsrc denotes the cluster resource, and config is the Spark configuration parameter set.

In this paper, we optimized the execution time of Spark applications by adjusting the Spark configuration parameters, which means that app, input, and rsrc are constants in Equation (5), config is the controlled variable, and Perf is the dependent variable based on the evaluation function. Therefore, this problem can be transformed into predicting the execution time of a Spark application given a set of configuration parameters.

**Figure 2:**
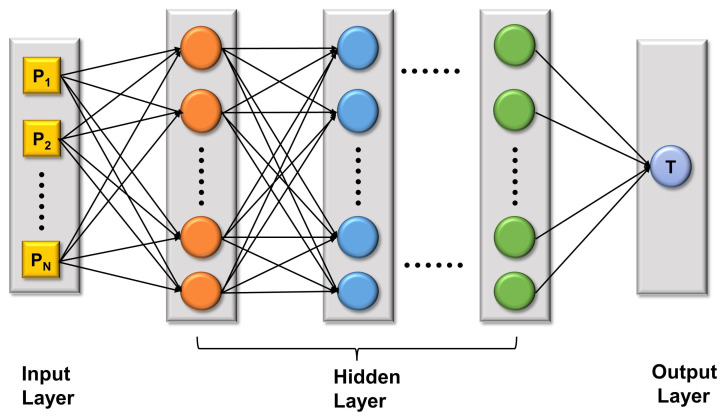
Deep neural network model.

We employed a deep neural network to build a Spark performance prediction model. The structure is shown in Figure 2. A deep neural network is mainly divided into three types of layers: the input layer, the hidden layer, and the output layer. These layers are fully connected, which indicates that any neuron in the *n*-th layer is connected with any neuron in the *n*+1-th layer. The connection relationship is expressed in Formula (6).

(6) $$y_{m}=f\left(\sum_{i=1}^{n}w_{im}x_{i}+b_{m}\right)$$

where *w* is the weight, *b* is the bias, $x_{i}$ is the neural unit, and f(.) is the activation function. In our deep neural network model structure, the input layer was the input of 16 Spark configuration parameters, the hidden layer was 3 layers (the number of neurons in each layer was 12, 8, and 4), and the output layer is the execution of the Spark application time. The activation function was rectified linear unit (ReLU). The definition of ReLU is f(x)=max(0,x), which is a popular activation function in neural networks. It is nonlinear and can be run backwards to minimize errors. During training, we applied an efficient optimizer, Adam, which iteratively updated the weights of the network on the basis of training data. At the same time, in order to render the model more accurate and with a stronger generalization ability, we also introduced Bayesian optimization to adjust the hyperparameters of our evaluation model, such as batch sizes, learning rates, and epochs.

### 3.3. Parameter Searching

The optimization problem of Spark configuration parameters in a discrete, finite search space can be modeled as a Markov decision process (MDP). An MDP consists of a quadruple $<S,A,P_{sa},R>$, where *S* is the finite state set, *A* is the finite motion set, $P_{sa}$ is the state transition probability, and *R* is the reward function [29]. After converting the Spark configuration parameter optimization problem into MDP, the configuration parameter set at the current moment is noted as the identifier of state *s*. Under this configuration parameter, the Spark application prediction time is used as the value of the state *s*, and each execution of an action is converted into another state.

Q-learning is a value-based reinforcement-learning algorithm that can solve the above MDP problem. The decision-making agent seeks the optimal strategy by maximizing the state action value function Q(*s*,*a*), uses S and A to construct a Q value table to store the value of Q(*s*,*a*), and selects the action that can obtain the maximal profit in the current state according to the Q value table in each step by adopting the ε-greedy strategy. The Q value update formula is shown in Equation (7).

(7) $$Q_{i+1}\left(s,a\right)=Q_{i}\left(s,a\right)+\alpha \left[r+\gamma \max_{a^{\prime }}Q_{i}\left(s^{\prime },a^{\prime }\right)-Q_{i}\left(s,a\right)\right]$$

where α is the learning rate, and γ is the conversion factor. When γ=0, only the immediate reward is considered. When γ = 1, the long-term reward is as important as the immediate reward. $s^{\prime }$ is the new state obtained after state transition, $a^{\prime }$ is the action selected according to the ε-greedy policy, and *r* is the immediate reward by taking action *a* from state *s* to state $s^{\prime }$.

In order to prevent the reinforcement-learning agent from conducting invalid searches among the parameter sets with poor performance, we improved the Q-learning algorithm, and did not set fixed starting and ending states in each epoch of the agent’s learning. The processing of the improved Q-learning algorithm is shown in Algorithm 1. initQtable(S,Q) means initializing the Q-Table and randomly selecting a set of configuration parameters from the parameter search space as the identifier of $s_{0}$, the predicted execution time of the Spark application with this configuration is used as the value of the state $s_{0}$, and all the corresponding values of Q($s_{0}$,*a*) are initialized to 0. bestSate records the best state at the current moment (the initial state is $s_{0}$). The outermost “For” loop represents the number of learning epochs, the “While” loop represents each epoch of learning, which starts from the best state at the current moment. chooseAction(s) uses the ε-greedy strategy to choose an action according to the Q-table. Each action means an update of configuration parameters and a transition to a new state $s^{\prime }$ after executing the action. If $s^{\prime }$ was not in the Q-table, we inserted it into Q-table and initialized all corresponding Q ($s^{\prime },a$) values to 0. updateQtable() was used to update the Q-table according to Formula (7). If state $s^{\prime }$ is better than the best state at the current moment, bestSate is updated. The ratio represents the ratio of performance improvement. If the ratio is less than 0.1, the current modified direction is not ideal, and  current $s^{\prime }$ is the termination state to end this epoch.
**Algorithm 1** The process of the improved Q-learning algorithm.  **Input:**        State List *S* = [$s_{0}$, $s_{1}$, $s_{2}$, …, $s_{n}$];        Qtable List *Q* = [$q_{0}$, $q_{1}$, $q_{2}$, …, $q_{n}$];  **Output:**        bestConf;  1: initQtable(*S*, *Q*)  2: bestState = $s_{0}$  3: **for** round from 1 to rounds **do**  4:     *s* = bestState //Store the best state  5:     **while** ratio > 0.1 **do**  6:         *a* = chooseAction( *s* ) //use the ε-greedy strategy to select the action  7:         $s^{\prime }$ = getOrCreateState(*s*, a) //if the state exists, return, does not exist, create and return  8:         *t*, $t^{\prime }$ = getTime(*s*, $s^{\prime }$) //obtain the predicted execution time of the Spark application corresponding to the status  9:         *r* = *t* − $t^{\prime }$ //use the time difference as a timely reward10:         updateQtable() // update Qtable according to Formula (7)11:         **if** $t^{\prime }$ < getTime( bestState ) **then**12:            bestState = $s^{\prime }$ //store the best state13:         ratio = (*t* − $t^{\prime }$)/*t* //compute the ratio of performance improvement14:         *s* = $s^{\prime }$15: **return** bestState

## 4. Experiments

In this section, we introduce the experimental environment (including Spark cluster configuration, Spark applications, and experimental data statistical methods), evaluation metrics for Spark performance prediction models, and the analysis of experimental results.

### 4.1. Experimental Setup

In order to evaluate the effectiveness of our proposed optimizer, we implemented the experiments on a cluster of six computing machines, each with Intel(R) Core(TM) i9-10900K CPU @ 3.70 GHz processor, 20 cores, 64 GB main memory, and 1 GigE Ethernet network. Our Spark cluster was based on CentOS Linux release 8.2.2004, JDK version 1.8, Apache Hadoop version 2.7.7, and Apache Spark 3.1.3.

To test the effectiveness of our method on different Spark application types, we chose WordCount, PageRank, KMeans, and TeraSort, provided by Hibench [27], as our benchmark applications. These four applications represent different workloads of Apache Spark. WordCount is CPU- and I/O-intensive, TeraSort is memory-intensive, and PageRank and KMeans are iteration-intensive.

After the collecting training data, we measured the execution time of each Spark application under a given configuration 9 times, and used the median as the real execution time of each application to eliminate bias caused by other factors such as the computer hardware and network.

### 4.2. Performance Metrics

In order to evaluate the quality of the Spark performance prediction model, we used the following evaluation metrics:

**Mean absolute error (MAE):** The average value of the absolute error can reflect the actual situation of the predicted value error. The smaller the MAE value is, the higher the model accuracy is. Assuming that the real values in the test set were $y_{1}$, $y_{2}$, $y_{3}$..., $y_{n}$ and the corresponding Spark performance model predicted values were $f_{1}$, $f_{2}$, $f_{3}$..., $f_{n}$, MAE is represented in Equation (8).

(8) $$\mathrm{MAE}=\frac{1}{n}\sum_{i=1}^{n}\left|y_{i}-f_{i}\right|$$

**Root mean square error (RMSE):** The standard deviation of the residuals (prediction errors). The smaller the RMSE value is, the higher the accuracy of the Spark performance prediction model is. The representation of RMSE is in Equation (9).

(9) $$\mathrm{RMSE}=\sqrt{\frac{1}{n}\sum_{i=1}^{n}{\left(y_{i}-f_{i}\right)}^{2}}$$

**R-squared ($R^{2}$):** The coefficient of determination can measure the fitness of one model. The closer the value of $R^{2}$ is to 1, the better the fit of the Spark performance prediction model is. The mean of the true values is $\bar{y}=\frac{1}{n}\sum_{i=1}^{n}y_{i}$. The representation of $R^{2}$ is:

(10) $$R^{2}=1-\frac{\sum_{i=1}^{n}{\left(y_{i}-f_{i}\right)}^{2}}{\sum_{i=1}^{n}{\left(y_{i}-\bar{y}\right)}^{2}}$$

**Mean absolute percentage error (MAPE):** The deformation (percentage value) of the MAE. The smaller the value of MAPE is, the better the accuracy of the Spark performance prediction model is. The representation of MAPE is shown in Equation (11):

(11) $$MAPE=\frac{100\%}{n}\sum_{i=1}^{n}\left|\frac{y_{i}-f_{i}}{y_{i}}\right|$$

### 4.3. Experimental Results

**Figure 3:**
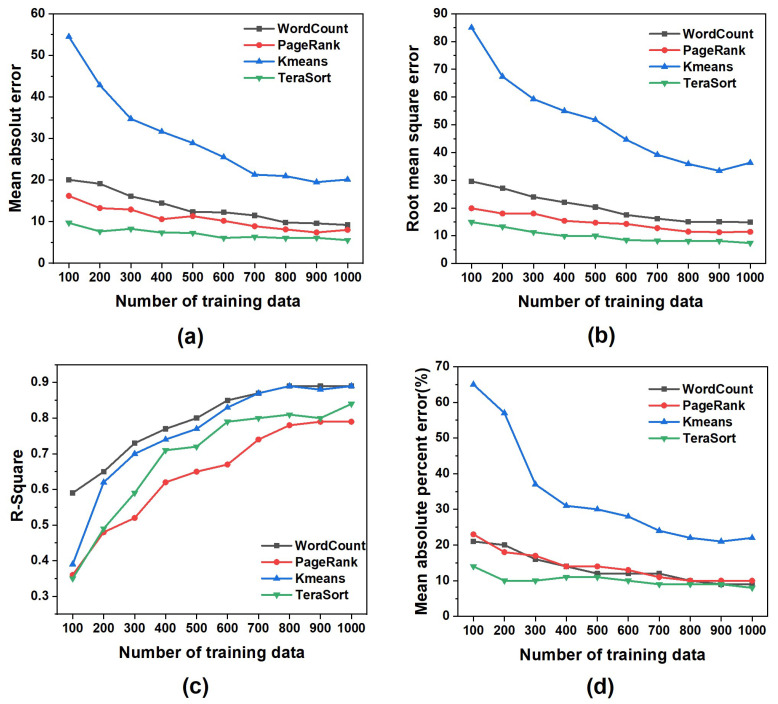
The number of training data is from 100 to 1000, and the evaluation indicators of different Spark application performance prediction models changed: (**a**) Mean absolute error. (**b**) Root mean square error. (**c**) R-squared. (**d**) Mean absolute percentage error.

**Relationship between the performance prediction model and the number of training data:** As with most machine-learning tasks, using more training data leads to better predictive models. However, in practice, since each training datum is collected by the Spark application execution in a real cluster environment, collecting too many training data increases the time cost. Therefore, we need to find a balance between various model evaluation indicators and the number of training data, that is, reduce the number of training data as much as possible under certain conditions. Figure 3 shows the changes in the evaluation indicators of different Spark application performance prediction models from 100 to 1000 training data. Figure 3a shows that, with the continuous increase in the number of training data, the MAE value of the prediction model of each Spark application gradually decreased, of which Kmeans varied most significantly. When the number of training samples reached 700, the MAE value was no longer significantly decreased. Figure 3b,d shows that the changing trends of RMSE and MAPE were similar to those in Figure 3a. Figure 3c shows that, with the continuous increase in the number of training data, the value of $R^{2}$ also rapidly increased, that is, the fitting degree of our model was improved. After the number of training data had reached 800, the value of $R^{2}$ tended to be stable. The above experimental analysis shows that it is better to build a performance prediction model for a Spark application when the number of training data is greater than 800.

**Figure 4:**
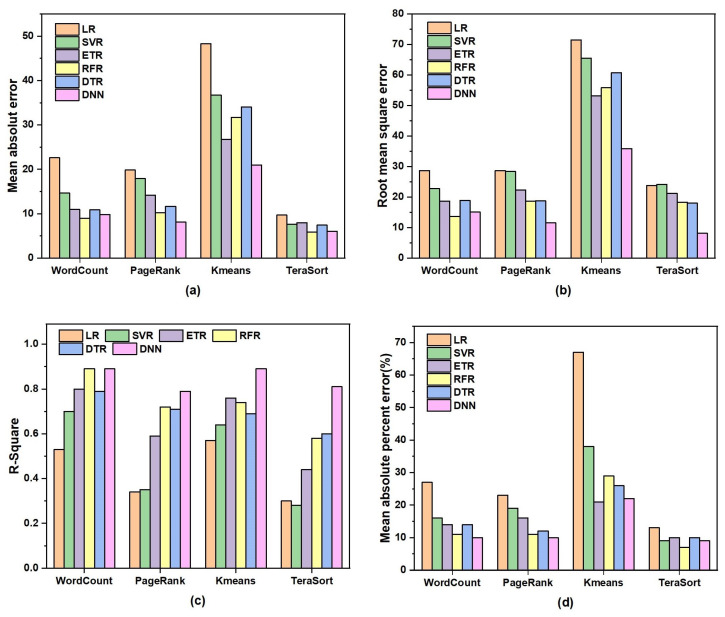
Performance comparison of deep neural-network models with other regression models on different Spark applications: (**a**) Mean absolute error. (**b**) Root mean square error. (**c**) R-squared. (**d**) Mean absolute percentage error.

**Performance comparison of deep neural network models and other regression models:** We compared Spark performance prediction models built with linear regression (LR), support vector machine regression (SVR), external tree regression (ETR), random forest regression (RFR), decision trees (DTR), and deep neural networks (DNNs). To fairly compare these methods, we used the same training environment, training dataset, and testing set as those of our proposed DNN method. Figure 4 shows the comparison of various evaluation metrics between different performance prediction models on four Spark applications. The DNN model showed significant improvement over the other models in all evaluation metrics for all Spark applications. For the Kmeans application in Figure 4b, the RMSE of the DNN model was reduced by 49.7%, 45.1%, 32.4%, 35.6%, and 40.8% compared to the LR, SVR, ETR, RFR, and DTR regression models, respectively. This is because Spark not only has a huge configuration parameter space, but the inter-relationship between configuration parameters is also very complex. Spark configuration parameter performance prediction models are definitely not linear, so LR performed the worst. For such high-dimensional and feature-related problems, shallow machine-learning methods such as SVR, ETR, and DTR are also not very effective. Deep learning can gradually learn through multiple networks, extract complex and effective features, and has higher prediction accuracy and generalization ability.

**Performance comparison between the improved Q-learning algorithm and other parameter search algorithms:**Table 2 shows the optimal values and search times for random search, simulated annealing, and the variant of the Q-learning algorithm on four Spark applications. In terms of optimal values, the random search algorithm performed the worst, with simulated annealing and improved Q-learning yielding very similar values. In terms of search time, the simulated annealing algorithm had the longest parameter search time, and the improved Q-learning algorithm had the shortest parameter. In the TeraSort application, the parameter search time of the improved Q-learning algorithm was reduced by 60.4% compared to the simulated annealing algorithm. The improved Q-learning algorithm could greatly reduce the time cost of parameter searching while obtaining better results, and achieved better time performance. This is because the agent of the improved Q-learning algorithm started from the best state at the current moment in each epoch of learning. If the next state was not better, the learning of this epoch was ended immediately, so as to prevent the agent from performing invalid searches among parameter sets with poor performance.

**Time performance overhead evaluation:** Our scheme consists of a performance prediction model and an improved Q-learning search algorithm. We applied the scheme proposed in this paper to four different types of Spark applications. Each type of application was conducted 9 times to search for the better configuration parameters and calculate the exact time cost, which is shown in Table 3. The time overhead of Spark configuration parameter optimization is about 61 s, which is within an acceptable range.

**Figure 5:**
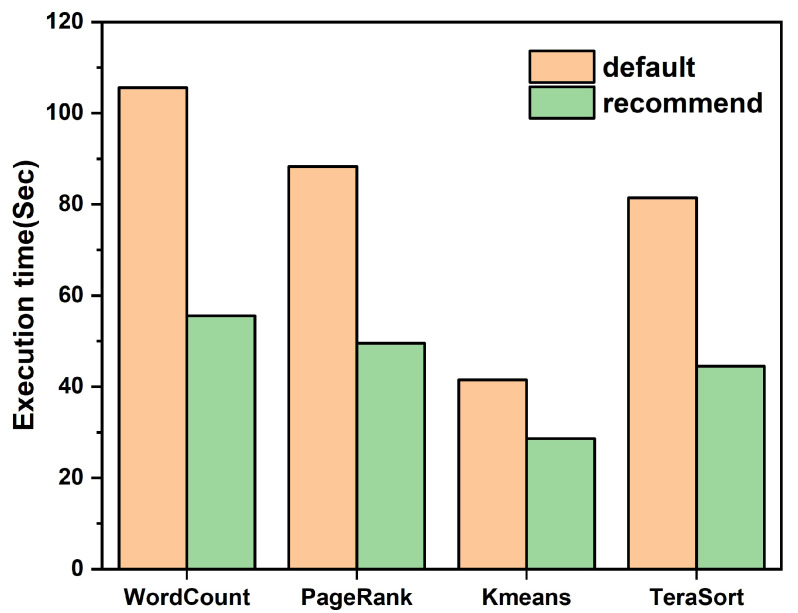
Execution time comparison between recommended configuration parameters and default configuration parameters on different Spark applications.

**Performance comparison between recommended configuration parameters and default configuration parameters:** By using our method, the recommended configuration parameters for four different types of Spark applications were obtained, as shown in Table 4. In order to avoid the contingency of the experiment, we submitted four types of Spark applications to the Spark cluster 9 times with the default and recommended configuration parameters, and obtained the median of execution time of Spark applications under the two configurations. The experimental results are shown in Figure 5. Compared with the default configuration, the recommended configuration for WordCount, PageRank, Kmeans, and TeraSort achieved performance improvement of 47%, 43%, 31%, and 45%, respectively, which shows that our Spark configuration parameter optimizer is effective in improving the performance of different types of Spark applications.

## 5. Conclusions

For Spark’s configuration parameter optimization problem, we employed a deep neural network to build a Spark performance prediction model, and designed an improved Q-learning algorithm as our Spark configuration parameter optimizer to search for better configuration parameters. The experimental results show that the Spark performance prediction model based on deep neural network is more accurate, and the improved Q-learning algorithm could greatly reduce the time cost of parameter searching while obtaining better results compared with the default configuration parameters for four different types of Spark applications.

Not only can the configuration parameters of the Spark application affect the performance, but the state of the Spark cluster (such as memory utilization, CPU utilization, and network bandwidth) can also influence the performance of the application. When CPU utilization is too high, it is better to allocate a small number of CPU cores to the application to reduce waiting time.

Our current work can only solve the optimizer problem for a single Spark application on a given Spark cluster. In future work, we aim to simultaneously optimize multiple Spark applications with the resource constraints of a Spark cluster.

## Figures and Tables

**Table 1:** Default Spark parameter values with range.

Spark Parameters	Function	Default	Range/Step
spark.executor.cores	Number of cores to use on each executor	1	1–8, 1
spark.executor.memory	Amount of memory to use per executor process	1	1–8, 1
spark.executor.instances	Number of executors	2	2–8,1
spark.driver.cores	Number of cores to use for the driver process	1	1–4, 1
spark.driver.memory	Amount of memory to use for the driver process	1 g	1–4, 1
spark.reducer.maxSizeInFlight	Maximal size of map outputs to fetch simultaneously from each reduce task	48 m	48–96, 8
spark.shuffle.compress	Whether to compress map output files	true	true, false
spark.shuffle.spill.compress	Whether to compress data spilled during shuffles	true	true, false
spark.shuffle.file.buffer	Size of the inmemory buffer for each shuffle file output stream	32 k	32–128, 16
spark.broadcast.blockSize	Size of each piece of a block for TorrentBroadcastFactory	4 m	4–24, 2
spark.broadcast.compress	Whether to compress broadcast variables before sending them	true	true, false
spark.memory.fraction	Fraction of (heap space—300 MB) used for execution and storage	0.6	0.3–0.8, 0.1
spark.memory.storageFraction	Amount of storage memory	0.5	0.3-0.8, 0.1
spark.rpc.message.maxSize	Maximal message size to allow in “control plane” communication	128 m	128–256, 32
spark.rdd.compress	Whether to compress serialized RDD partitions	false	true, false
spark.io.compression.codec	The codec used to compress internal data such as RDD partitions, event logs, broadcast variables, and shuffle outputs	lz4	Lz4, snappy

**Table 2:** Performance comparison of the improved Q-learning algorithm and other search algorithms on different Spark applications.

Algorithms	WordCount		PageRank		Kmeans		TeraSort	
	Value (s) ^1^	Time (ms) ^2^	Value (s) ^1^	Time (ms) ^2^	Value (s) ^1^	Time (ms) ^2^	Value (s) ^1^	Time (ms) ^2^
Rand	51.9	653.0	50.5	463.8	26.2	589.4	42.6	818.6
Simulated Annealing	50.9	830.6	49.4	835.8	25.8	854.0	41.8	905.4
Q-learning	50.2	463.8	49.6	435.0	26.1	296.4	42.5	358.0

^1^ The optimal value searched by the algorithm; ^2^ time indicates the time spent by the algorithm to search for the optimal value.

**Table 3:** The time overhead of better configuration parameter solving for different types of Spark applications.

Spark Application	Time (s)
WordCount	61.571
PageRank	61.402
Kmeans	61.543
TeraSort	61.345

**Table 4:** Default parameter values and recommended parameter values for different Spark applications.

Parameter	Default	Recommend			
		WordCount	PageRank	Kmeans	TeraSort
spark.executor.cores	1	8	6	7	5
spark.executor.memory	1 g	5 g	4 g	4 g	7 g
spark.executor.instances	2	4	5	5	6
spark.driver.cores	1	2	3	3	3
spark.driver.memory	1 g	3 g	1 g	2 g	3 g
spark.reducer.maxSizeInFlight	48 m	48 m	72 m	56 m	36 m
spark.shuffle.compress	true	true	true	false	true
spark.shuffle.spill.compress	true	true	false	true	false
spark.shuffle.file.buffer	32 k	48 k	96 k	48 k	112 k
spark.broadcast.blockSize	4 m	4 m	10 m	6 m	6 m
spark.broadcast.compress	true	true	false	true	false
spark.memory.fraction	0.6	0.4	0.7	0.5	0.4
spark.memory.storageFraction	0.5	0.6	0.5	0.5	0.4
spark.rpc.message.maxSize	128 m	256 m	128 m	192 m	160 m
spark.rdd.compress	false	true	false	false	true
spark.io.compression.code	lz4	lz4	snappy	lz4	snappy

## Data Availability

Dataset link: https://github.com/Intel-bigdata/HiBench (accessed on 26 June 2022).
